# Combining Fluorescence and Magnetic Resonance Imaging in Drug Discovery—A Review

**DOI:** 10.3390/ph19010056

**Published:** 2025-12-26

**Authors:** Barbara Smolak, Klaudia Dynarowicz, Dorota Bartusik-Aebisher, Gabriela Henrykowska, David Aebisher, Wiesław Guz

**Affiliations:** 1 Department of Diagnostic Imaging and Nuclear Medicine, Faculty of Medicine, University of Rzeszów, 35-310 Rzeszów, Poland; bsmolak@ur.edu.pl; 2 Department of Biochemistry and General Chemistry, Faculty of Medicine, University of Rzeszów, 35-310 Rzeszów, Poland; kdynarowicz@ur.edu.pl (K.D.); dbartusikaebisher@ur.edu.pl (D.B.-A.); 3 Department of Epidemiology and Public Health, Faculty of Medicine, Medical University of Lodz, Tadeusza Kosciuszki 4, 90-419 Lodz, Poland; gabriela.henrykowska@umed.lodz.pl; 4 Department of Photomedicine and Physical Chemistry, Faculty of Medicine, University of Rzeszów, 35-310 Rzeszów, Poland

**Keywords:** fluorescence, magnetic resonance imaging (MRI), multimodal imaging, hybrid probes, drug discovery, pharmacokinetics, nanotechnology in medicine

## Abstract

Drug discovery is a complex and multi-stage process that requires advanced analytical technologies capable of accelerating preclinical evaluation and improving the precision of therapeutic design. The combination of fluorescence and magnetic resonance imaging (MRI) within multimodal imaging plays an increasingly important role in modern pharmacokinetics, integrating the high molecular sensitivity of fluorescence with the non-invasive anatomical visualization offered by MRI. Fluorescence enables real-time monitoring of cellular processes, including drug–target interactions and molecular dynamics, whereas MRI provides detailed structural information on tissues without exposure to ionizing radiation. Hybrid probes—such as superparamagnetic iron oxide nanoparticles (SPIONs) functionalized with near-infrared (NIR) fluorophores or gadolinium-based complexes linked to optical dyes—enable simultaneous acquisition of molecular and anatomical data in a single examination. These multimodal systems are being explored in oncology, neurology, and cardiology, where they support improved visualization of tumor biology, amyloid pathology, and inflammatory processes in vascular disease. Although multimodal imaging shows great promise for enhancing pharmacokinetic and pharmacodynamic studies, several challenges remain, including the potential toxicity of heavy-metal-based contrast agents, limited tissue penetration of fluorescence signals, probe stability in vivo, and the complexity and cost of synthesis. Advances in nanotechnology, particularly biodegradable carriers and manganese-based MRI contrasts, together with the integration of artificial intelligence algorithms, are helping to address these limitations. In the future, fluorescence–MRI hybrid imaging may become an important tool in personalized medicine, supporting more precise therapy planning and reducing the likelihood of clinical failure.

## 1. Introduction

The discovery of new drugs is a multi-stage, complex, and costly process that requires the integration of advanced technologies to enhance therapeutic efficacy and shorten the time to market for innovative products. Modern pharmaceutics faces a growing number of diseases with complex and multifactorial etiologies, such as cancer, neurodegenerative disorders, and autoimmune diseases. In this context, advanced imaging techniques play a key role in accelerating drug research by enabling precise analysis of interactions between drug molecules and biological structures at both the molecular and cellular levels [1,2,3]. Imaging techniques, including fluorescence imaging and magnetic resonance imaging (MRI), are crucial in the development of modern pharmaceuticals, as they enable real-time visualization of biological processes and the assessment of drug candidate efficacy in preclinical models. Fluorescence imaging allows for molecular dynamics analysis with exceptionally high sensitivity, whereas MRI provides high-resolution, anatomically precise images of tissues, making these techniques complementary diagnostic tools. The integration of these methods within multimodal imaging approaches offers broad prospects for identifying therapeutic targets, monitoring drug bioavailability, and comprehensively assessing drug effects in living organisms [4,5,6]. The aim of this paper is to present the synergistic effects of fluorescence imaging and magnetic resonance imaging (MRI) in the context of drug discovery and design. The paper discusses the theoretical foundations of both techniques, their applications in pharmaceutical research, and the benefits of their integration. Particular attention is given to practical examples of their use, technological challenges, and future development prospects in this rapidly evolving field.

## 2. Multimodal Imaging Techniques in Molecular Analysis and Drug Discovery

### 2.1. The Phenomenon of Fluorescence and Its Importance in Molecular Analysis

Upon absorption of a photon, an atom or molecule can transition from its ground state to a higher-energy state. In more complex molecular systems, the return to the ground state does not always occur in a single step, as additional energy levels may exist between the excited and ground states. In such cases, radiation emission occurs in stages, and the emitted photons have lower energy and longer wavelengths than the absorbed photons. When absorption occurs in the ultraviolet range and emission occurs in the visible region of the spectrum, this phenomenon is known as fluorescence. This process primarily involves molecules with complex electronic structures, and the associated relaxation phenomena include a series of internal transitions characteristic of such systems [7].

The wavelength of fluorescence depends directly on the energy difference between the electronic states of a molecule. Consequently, the frequency and nature of the emitted radiation are characteristic features of a given substance, which explains why many chemical compounds exhibit natural fluorescence. This property makes fluorescence an exceptionally sensitive tool for chemical identification in both qualitative and quantitative analyses. It is widely used to detect trace amounts of substances, monitor their metabolism in living organisms, and track biochemical changes in real time [7]. Detection selectivity depends on the appropriate choice of excitation source, which should be as monochromatic as possible, as well as on the properties of the solvent, which must not emit in the same spectral range. In simple cases, the color or intensity of the emission may be sufficient for compound identification, whereas in more advanced analyses, fluorescence spectrometers are employed to enable precise measurements of emission wavelength and intensity [7].

**Figure 1:**
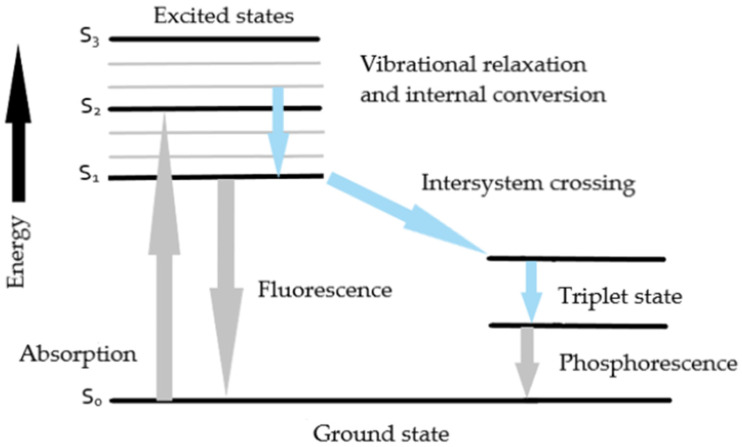
Jabłoński diagram showing the mechanism of fluorescence and phosphorescence.

Fluorescence is a quantum-mechanical phenomenon resulting from electronic transitions occurring in molecules, atoms, and ions. Upon absorption of a photon with sufficient energy, an electron is promoted from the ground state to a higher energy level in accordance with the principles of quantum mechanics [8]. This excited state is short-lived, and the electron subsequently returns to the ground state, releasing excess energy in the form of a photon. Part of the excitation energy may be dissipated through vibrational relaxation or non-radiative processes, resulting in a reduction in the energy of the emitted light. The energy difference between the absorbed and emitted photons, known as the Stokes shift, is one of the key parameters characterizing the fluorescent properties of a molecule [7,8]. The phenomenon of fluorescence is commonly illustrated using the Jablonski diagram, which depicts transitions between the ground state (S_0_) and excited states (S_1_, S_2_, etc.), along with processes such as absorption, vibrational relaxation, internal conversion, and fluorescence emission [7,8]. This diagram (Figure 1) provides a fundamental framework for interpreting luminescence phenomena and is widely used to describe the dynamics of molecular energy transitions.

The mechanism of fluorescence begins with the absorption of light energy by a molecule known as a fluorophore. The photon energy must be sufficient to promote an electron from its ground state to an excited state. This excited state is characterized by a lifetime ranging from several picoseconds to several nanoseconds, during which non-radiative processes may occur, such as internal relaxation or interactions with the surrounding environment, including collisions with other molecules. These processes lead to energy losses in the form of heat and result in a reduction in the energy of the emitted radiation. The key step in fluorescence is the emission of a photon accompanying the electron’s return to the ground state, which occurs with a slight delay relative to absorption. The Stokes shift reflects these energy losses and remains one of the most important parameters describing fluorophore behavior [7,8,9].

The properties of fluorescent emission are strongly influenced by the molecular environment. Fluorescence quenching processes arise from interactions between the fluorophore and other molecules, leading to the loss of excitation energy without photon emission. Quenching may be dynamic, resulting from collisions between molecules in the excited state, or static, involving the formation of non-emissive complexes [7,8,9]. A thorough understanding of fluorescence mechanisms has enabled the development of modern research techniques such as fluorescence spectroscopy and fluorescence microscopy. These methods allow the analysis of biological and chemical processes at the molecular level, enable precise visualization of structures in living cells and tissues, and facilitate real-time tracking of biomolecular dynamics. Consequently, fluorescence-based techniques have found widespread application in cancer research, neurobiology, molecular diagnostics, and the design of targeted therapies. Owing to its high sensitivity, selectivity, and capability for live-cell observation, fluorescence remains a key tool in modern experimental pharmacology and biomedical imaging [7,8,9].

#### 2.1.1. Fluorescent Markers in Biological Research

Fluorescent markers are among the most important tools in modern experimental biology and biomedicine. They are chemical compounds or molecular structures capable of emitting light when excited by radiation of the appropriate wavelength, allowing for their widespread use in biological research. The unique ability of these molecules to emit radiation in the visible or near-infrared range enables precise tracking of biological processes at the cellular and molecular levels, often in real time. As a result, fluorescent markers have become the basis for modern imaging methods, enabling the visualization of structures and processes occurring in living cells and tissues in vivo [10,11].

The most commonly used fluorescent markers include organic fluorophores, fluorescent proteins—such as green fluorescent protein (GFP) and its numerous mutants—and dyes emitting in the near-infrared range, including indocyanine green (ICG). Each of these groups is characterized by specific physicochemical and biological properties, allowing for their precise tailoring to a specific research goal. Organic fluorophores such as fluorescein, rhodamine, and ICG are distinguished by their high emission intensity, broad wavelength range, and chemical modification capabilities. This allows them to be conjugated to proteins, nucleic acids, or nanostructures, making them versatile tools in molecular biology and pharmacology [12,13,14].

Fluorescent proteins—especially GFP and its derivatives—have revolutionized biological research, enabling the direct observation of gene expression and protein localization in cells and model organisms such as Mus musculus and Danio rerio [15]. In turn, near-infrared fluorophores, particularly ICG, are exceptionally useful for in vivo imaging. They emit radiation in a spectral range where absorption and scattering of light in tissues are minimal, allowing for high-contrast images with significantly greater penetration depth than fluorophores emitting in the visible spectrum [16]. ICG plays a special role in NIR imaging because it minimizes interference with tissue autofluorescence, enabling a stable signal even in complex biological environments. In practice, it is used in vascular imaging, tissue perfusion assessment, drug distribution monitoring, and fluorescence-guided surgery. Its photophysical properties—high quantum yield, good aqueous solubility, and low toxicity—make it one of the most commonly used clinical markers in diagnosis and therapy [17,18].

Modern fluorescent markers are characterized by very high sensitivity and, in many cases, enable the detection of single molecules in a biological sample. However, to obtain reliable and reproducible results, careful selection of the marker is necessary, including its compatibility with the system being studied, resistance to photobleaching, and minimal interference with the natural autofluorescence of tissues. An incorrectly selected fluorophore can lead to reduced signal intensity or misinterpretation of the obtained data [19].

In the context of drug research, fluorescent markers—especially those like ICG—are an extremely useful tool for tracking the distribution of active substances in cells and tissues, assessing their bioavailability, and conducting preliminary toxicity analysis. They thus enable faster verification of potential drug candidates and shorten the time required for their further optimization. Thanks to the development of successive generations of fluorophores with increased photostability, selectivity, and biocompatibility, fluorescence techniques remain one of the most dynamically developing areas of contemporary bioanalytics and experimental pharmacology [17,20].

#### 2.1.2. Applications of Fluorescence in Molecular Analysis

Fluorescence is a key tool in modern molecular analysis, enabling the study of biological structures and processes with exceptional precision and sensitivity. Fluorescence-based techniques enable the observation of interactions between molecules, the analysis of cellular dynamics, and the monitoring of changes in the chemical microenvironment at nano- and micrometric scales. The characteristic wavelengths of light emitted by fluorophores enable the tracking of processes such as ligand binding, protein conformational changes, and the transport of molecules within cells. The high selectivity of this emission enables the simultaneous monitoring of multiple biological processes using different dyes, paving the way for advanced multiparameter studies and correlational analyses encompassing various levels of cellular organization [21,22]. One of the most important applications of fluorescence is fluorescence microscopy, which has significantly transformed the way cellular structures are observed. The development of techniques such as confocal microscopy and superresolution microscopy has enabled resolution exceeding the diffraction limits of classical optical microscopy [23,24]. These methods have enabled detailed visualization of subcellular structures—cell membranes, organelles, and the cytoskeleton—as well as real-time analysis of their interactions. The FRET technique plays a crucial role in cell signaling research, enabling the analysis of energy transfer between two fluorophores. This method allows for precise determination of the distance between fluorophores at the nanometer scale, thus enabling the study of protein–protein interactions that underlie many regulatory processes in cells [25,26].

Modern fluorescence techniques also include fluorescence lifetime imaging microscopy (FLIM), which allows for the assessment of changes in the molecular environment regardless of signal intensity. Fluorescent biosensors are also being developed, exploiting the variations in fluorophore emission properties to detect parameters such as pH, redox potential, and the presence of metal ions. Combining these methods with magnetic resonance imaging (MRI) or optoacoustic techniques leads to the creation of advanced multimodal imaging systems that combine the sensitivity of fluorescence with the high anatomical and functional resolution of other modalities [27,28].

Fluorescence is also widely used in analytical techniques, such as fluorescence spectroscopy, which enables the quantitative determination of substance concentrations in biological samples both in vitro and in vivo. This method allows, among other things, monitoring the concentration of calcium, hydrogen, and oxygen ions in cells, providing key information about physiological processes such as intracellular signaling, metabolism, and muscle contraction. In pharmacology and drug discovery research, fluorescence plays a significant role in tracking the distribution of molecules within cells, analyzing their binding to target receptors, and assessing bioavailability and metabolism [29,30].

From a photophysical perspective, fluorescence properties are described by quantitative parameters such as fluorescence quantum yield (ΦF), excited-state lifetime (τF), and Stokes shift (Δλ). High quantum yield and photochemical stability of fluorophores are essential for obtaining reliable and reproducible results, especially in biological applications. These parameters provide information about the dynamics of electronic transitions, emission efficiency, and the influence of the environment on the fluorophore, which is fundamental for the design of fluorescent probes and markers [31]. However, the high sensitivity of fluorescence techniques requires carefully planned experiments. This requires the proper selection of the fluorophore, optimization of excitation and emission conditions, and minimization of the influence of tissue autofluorescence, which can interfere with the measurement. Despite these limitations, fluorescence remains an indispensable tool for molecular analysis, combining exceptional detection sensitivity with the ability to observe biological processes in real time. Consequently, it is one of the pillars of the development of modern diagnostic, therapeutic, and research methods in biomedical sciences. Table 1 presents selected key quantitative parameters describing fluorescence, their definitions and their importance in molecular analysis.

#### 2.1.3. Advantages and Limitations of Fluorescence Techniques

Fluorescence techniques are among the fundamental tools of modern biological and pharmaceutical research, offering unique opportunities for analyzing molecular processes with exceptional sensitivity and specificity. Their effectiveness stems from the combination of high detection sensitivity, the ability to differentiate chemical structures, and the ability to observe biological processes in real time. This allows for the precise monitoring of changes occurring in cells and tissues, providing the foundation for many imaging methods and biophysical analyses [32]. One of the greatest advantages of fluorescence is its very high sensitivity, enabling the detection of even single molecules in complex biological systems. This enables the analysis of low concentrations of biomolecules such as proteins, nucleic acids, and metabolites, while maintaining high measurement precision. Fluorescence also allows for the tracking of dynamic biological processes, such as ligand–receptor interactions, cell migration, and the reorganization of cytoskeletal elements. The ability to conduct real-time observations makes fluorescence techniques widely used in studies of regulatory and signaling mechanisms in living cells [33,34].

Another advantage of fluorescence methods is their high spatial resolution, particularly in confocal microscopy and superresolution techniques, which enable precise mapping of subcellular structures and analysis of their interactions. The emission specificity characteristic of different fluorophores is also crucial, enabling the simultaneous monitoring of multiple processes in a single experimental system and significantly increasing the potential of multiparameter analyses [35].

Despite numerous advantages, fluorescence techniques also have certain limitations that can affect the reliability of the obtained results. One of the most serious problems is the autofluorescence of biological tissues, which generates a background signal that can interfere with fluorophore emission and complicate data interpretation, especially in tissues with complex structures. Another challenge is fluorophore photodegradation (photobleaching), which is the gradual loss of light emission due to prolonged or intense exposure to excitation radiation. This phenomenon limits observation time and measurement repeatability, particularly in studies requiring repeated or continuous monitoring. Limited light penetration in biological tissues poses another challenge, particularly for fluorophores emitting in the visible spectrum, which reduces the effectiveness of imaging structures deeper within living organisms. An additional factor determining experimental success is the need for careful selection of fluorescent markers, considering their photostability, potential toxicity, compatibility with a given biological system, and minimizing their impact on physiological processes [35,36].

**Figure 2:**
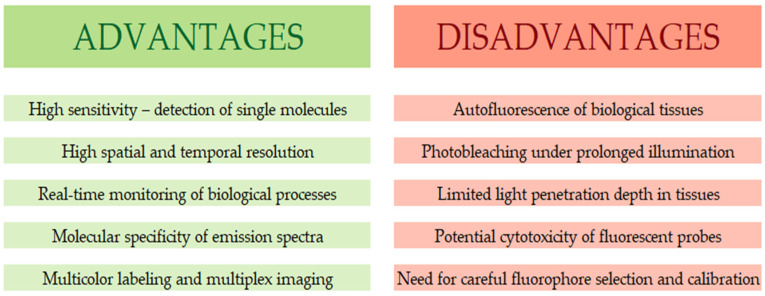
Summary of the main advantages and limitations of fluorescence techniques.

However, the appropriate selection of fluorophores and optimization of experimental parameters allow for the full potential of fluorescence techniques. The ongoing development of new generations of markers, particularly fluorophores emitting in the near-infrared range, characterized by increased photostability and high biocompatibility, is systematically expanding the possibilities of biological and pharmacological imaging. Consequently, fluorescence remains one of the most dynamically developing areas of modern bioanalytics and forms the foundation of many contemporary diagnostic and research applications. Figure 2 shows main advantages and limitations of fluorescence techniques.

### 2.2. Magnetic Resonance Imaging (MRI) in Imaging Biological Structures and Processes

Magnetic resonance imaging (MRI) is one of the most advanced diagnostic imaging techniques, widely used in clinical medicine, scientific research, and pharmaceuticals. Based on the phenomenon of nuclear magnetic resonance, this method enables the acquisition of images with high spatial resolution, enabling detailed assessment of biological structures and analysis of their function. Unlike techniques using ionizing radiation, MRI is completely non-invasive and safe for patients, and its ability to detect subtle pathological changes makes it widely used in both diagnostics and therapy monitoring [37,38,39].

MRI offers extensive imaging capabilities for tissue morphology and function, enabling the assessment of physiological processes such as perfusion, diffusion, and neuronal activity. For this reason, this technique is indispensable in researching the pathogenesis of cancer, neurodegenerative diseases, and cardiovascular diseases. Its importance extends beyond standard clinical diagnostics, however. MRI plays a key role in pharmacokinetic and pharmacodynamic studies, enabling non-invasive monitoring of drug distribution in the body, assessment of drug metabolism, and the impact on the function of individual tissues [38,39].

The development of magnetic resonance imaging technology has led to revolutionary changes in neuroscience with the introduction of functional MRI (fMRI). This technique enables non-invasive mapping of brain activity in real time, allowing for the analysis of cognitive processes, emotional responses, and neurological pathologies. Therefore, fMRI provides key information in the study of the mechanisms of neurodegeneration, mental disorders, and functional brain organization [40].

This chapter provides an overview of the physical basis of MRI, the architecture of imaging systems, and the most important directions for the development of this technology. The role of contrast agents, clinical and pharmaceutical applications, and the main benefits and limitations of MRI in drug development are also discussed.

#### 2.2.1. Physical Basics of Magnetic Resonance Imaging

**Figure 3:**
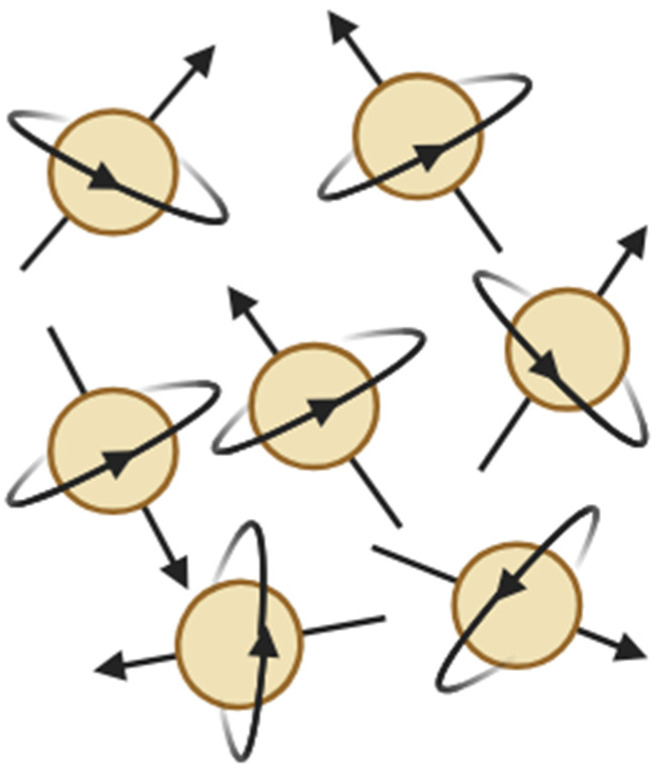
Free protons—lack of spin alignment in the state without a magnetic field.

Magnetic resonance imaging (MRI) is based on the phenomenon of nuclear magnetic resonance (NMR), which describes the interaction of atomic nuclei possessing non-zero nuclear spin with an external magnetic field and radiofrequency (RF) electromagnetic radiation [41,42,43,44,45]. Among biologically relevant nuclei, the hydrogen nucleus (^1^H) is of primary importance due to its high natural abundance and large gyromagnetic ratio. Nuclear spin is a fundamental quantum-mechanical property of nuclei, analogous to mass or electric charge, and does not correspond to classical mechanical rotation of the nucleus around its axis. Although the spin angular momentum exhibits mathematical similarities to classical angular momentum, it has no direct classical analog. Nevertheless, for didactic purposes, spin is often heuristically associated with rotation, as it gives rise to a magnetic dipole moment μ proportional to the nuclear spin angular momentum I [46]. Each hydrogen nucleus therefore behaves as a microscopic magnetic dipole. In the absence of an external magnetic field, the orientations of these dipoles are randomly distributed, resulting in zero net magnetization within the sample (Figure 3).

##### Spin Polarization and Macroscopic Magnetization

**Figure 4:**
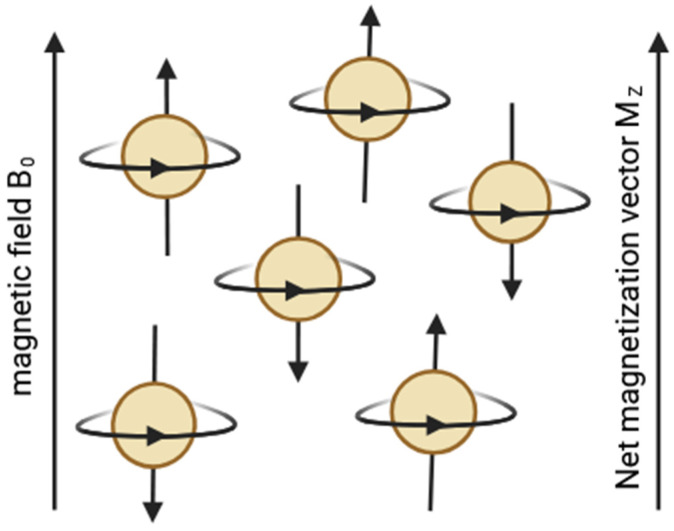
Spinning protons in a magnetic field—at 0 K, all spins occupy a lower energy state.

When a sample is placed in an external static magnetic field B_0_, nuclear magnetic moments interact with the field, leading to Zeeman splitting into discrete energy levels. For spin-½ nuclei such as protons, two energy states are possible: one aligned parallel to the field (lower energy) and one antiparallel (higher energy). At temperatures close to absolute zero (0 K) (Figure 4), all spins occupy a lower-energy state.

**Figure 5:**
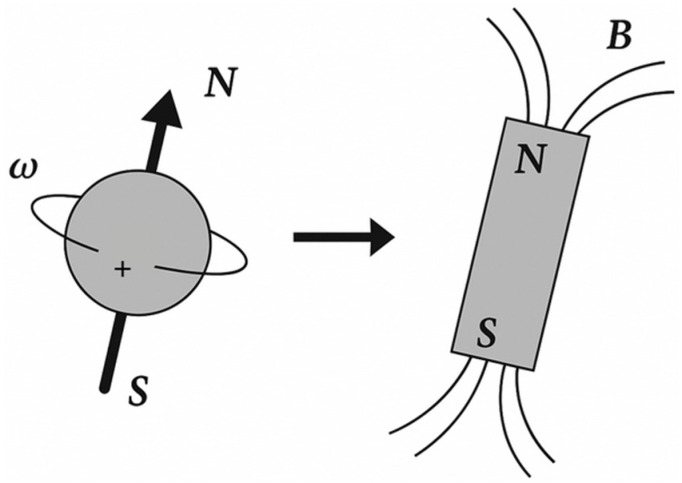
Proton spin and field magnetization.

According to the Boltzmann distribution, even at physiological temperatures (~300 K), the population difference between these two states is very small—on the order of one excess spin per several thousand to tens of thousands of nuclei, depending on field strength. Despite this small imbalance, the collective effect of a large number of spins gives rise to a measurable macroscopic magnetization vector M_0_ aligned with the direction of the external magnetic field (Figure 5) [46].

It is important to note that spin polarization occurs even in very weak magnetic fields, including the Earth’s magnetic field. However, the resulting Larmor frequencies lie in the kHz range, and the induced signals are extremely weak, requiring highly sensitive detection hardware. Clinical MRI systems operate at much stronger fields (typically 1.5–7 T), producing resonance frequencies in the MHz range, which significantly improves signal-to-noise ratio and imaging feasibility.

##### Larmor Precession

In a magnetic field, nuclear magnetic moments do not remain static but undergo precessional motion around the direction of B_0_. The angular frequency of this motion, known as the Larmor frequency, is given by:

$$\omega_{0}=-\gamma B_{0}$$

where

ω_0_—angular Larmor frequency,

γ—gyromagnetic ratio (for protons: 42.58 MHz/T),

B_0_—magnetic field strength.

The negative sign reflects the fact that nuclear magnetic moments precess in a direction determined by the sign of γ, a detail that becomes important in rigorous quantum-mechanical descriptions and rotating-frame formalism.

**Figure 6:**
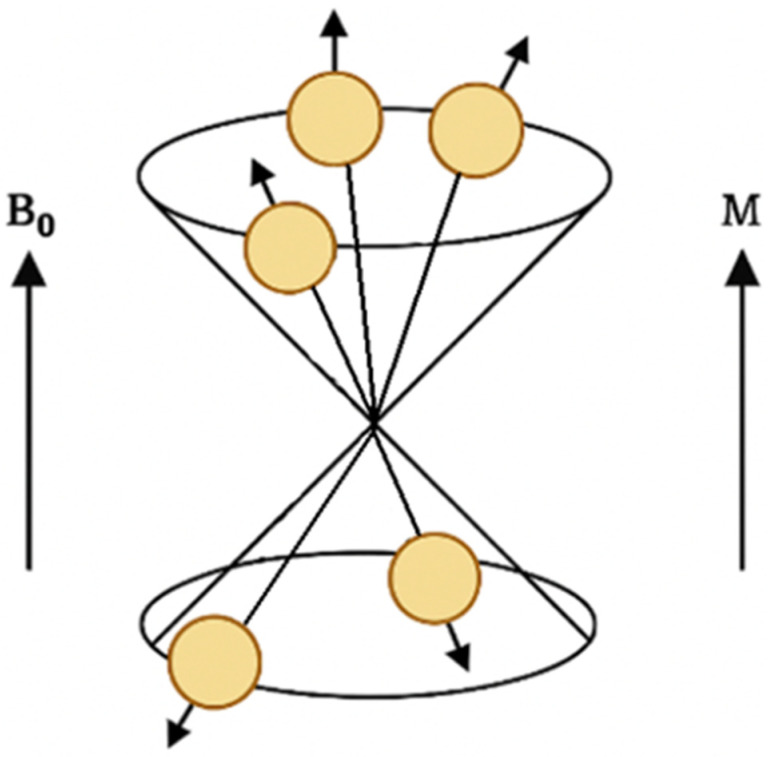
The magnetization phenomenon.

Figure 6 shows the magnetization phenomenon.

##### RF Excitation and Signal Detection

When an RF pulse is applied at the Larmor frequency, it induces transitions between the Zeeman energy levels and coherently perturbs the macroscopic magnetization vector away from its equilibrium orientation. In the rotating frame of reference, this corresponds to a rotation of the magnetization vector by a defined flip angle (e.g., 90° or 180°), determined by the amplitude and duration of the RF pulse. After termination of the RF pulse, the magnetization evolves freely under the influence of the static magnetic field and spin–spin interactions. Importantly, the detected NMR signal does not arise from energy “emission” by relaxing spins. Instead, the time-dependent precession of the transverse magnetization induces an electromotive force in the receiver coil, in accordance with Faraday–Lenz law of electromagnetic induction. This induced voltage constitutes the free induction decay (FID) signal, which decays over time due to relaxation processes and magnetic field inhomogeneities [46].

##### Relaxation Mechanisms

The return of the nuclear spin system to thermal equilibrium is described by two fundamental relaxation processes:

- Longitudinal (spin–lattice) relaxation (T_1_)—the recovery of the magnetization component parallel to B_0_, governed by energy exchange between the spin system and its molecular environment.
- Transverse (spin–spin) relaxation (T_2_)—the loss of phase coherence among spins in the transverse plane due to local magnetic field fluctuations and spin–spin interactions.

These relaxation times are tissue-specific and depend on molecular mobility, water content, and microstructural properties. Differences in T_1_ and T_2_ values form the physical basis of contrast in MRI images, enabling differentiation between various tissue types. By appropriate manipulation of RF pulse timing, flip angles, and repetition parameters, MRI pulse sequences can selectively emphasize T_1_, T_2_, or proton density (PD) contrast. For example, a 90° excitation pulse followed by a 180° refocusing pulse generates a spin-echo, compensating for static field inhomogeneities and allowing more accurate measurement of T_2_ relaxation. In advanced techniques such as magnetic resonance spectroscopy (MRS), detailed analysis of the FID signal in the frequency domain enables identification of chemical shifts associated with specific metabolites, including N-acetylaspartate (NAA), choline, and creatine. This provides valuable metabolic information complementary to conventional MRI in neurological and oncological applications.

#### 2.2.2. Construction of MRI Systems

The architecture of a magnetic resonance imaging (MRI) system includes integrated components designed to generate, modulate, and detect magnetic signals with the highest possible precision. The central element of every MRI scanner is a superconducting magnet responsible for generating a stable, uniform magnetic field, typically 1.5–3 T in clinical scanners and up to 7–14 T in research systems. The use of such strong magnetic fields enables ultrahigh-resolution images, enabling the visualization of submicrometer structures and precise characterization of biomaterials [47].

Superconducting electromagnets made from materials such as niobium–titanium alloy (NbTi) remain the standard due to their ability to generate strong, uniform, and stable magnetic fields at relatively low energy. This is crucial for image quality and a high signal-to-noise ratio (SNR) in clinical images [48].

In parallel to superconducting electromagnets, classical resistive electromagnets continue to play a significant role in specific MRI setups. Resistive electromagnets are based on large copper coils that generate a magnetic field under the influence of a direct current without the use of ferromagnetic materials [49]. Their main advantages are their relatively low construction cost and the absence of cryogenic cooling, which simplifies installation and operation. However, their use in high-field scanners is limited by significant power consumption, heat generation, and difficulty maintaining field homogeneity above 0.3–0.5 T, which has limited their practical use to low- and medium-field devices.

The magnet windings, made of niobium–titanium (NbTi) composites, are cooled with liquid helium to temperatures below −269 °C, which induces their superconductivity. Superconductivity eliminates energy losses and enables long-term, stable operation of the system without the need for a constant electrical supply. The stability of the field generated by the magnet is crucial for imaging quality, as minimal fluctuations can affect the accuracy of spatial encoding and image reconstruction [47].

Another important element of the system is the RF (radiofrequency) transmitting and receiving coils, which perform a dual function: they generate short pulses of electromagnetic waves that excite the spins of atomic nuclei and record relaxation signals after excitation. Depending on the application, surface coils are distinguished and characterized by very high sensitivity in the examination of superficial structures, and volume coils ensure uniform coverage of the entire examined volume, making them particularly useful in imaging the brain, heart, or abdominal organs. The RF system includes a pulse generator, a power amplifier (up to 35 kW), and receiving modules responsible for signal analysis, enabling precise emission and recording of data across a wide range of parameters [47].

An integral part of an MRI scanner is gradient electromagnets arranged orthogonally with respect to the X, Y, and Z axes. Their function is to introduce controlled inhomogeneities in the magnetic field, enabling spatial signal encoding and the reconstruction of high-fidelity three-dimensional images. Gradient coils operate in modes requiring rapid changes in field strength, so their design must consider resistance to mechanical stress and thermal effects. A characteristic side effect of gradient coil operation is acoustic noise, which in high-field systems can exceed 130 dB. For this reason, modern scanners are equipped with vibration-damping systems and acoustic insulation to reduce the perceived noise level [47].

In recent years, the literature and technical reviews have strongly highlighted a resurgence of interest in low-field MRI systems [50] (<0.5 T), which often utilize both resistive electromagnets and permanent magnet configurations equipped with appropriately ordered magnetic arrays (e.g., Halbach arrays) [51] to generate stable static fields without the costs associated with cryogenic cooling. Recent reviews have shown that this approach—combined with modern image reconstruction methods, advanced RF antennas, and machine learning algorithms—significantly improves the clinical utility of low-field MRI systems, especially in mobile, point-of-care applications and in environments with limited medical infrastructure and where installation costs are critical [52]. A key advantage of such solutions is the reduction in operational and infrastructure costs: the absence of large cryocoolers eliminates the need for liquid helium, and simplified magnetic enclosures reduce room shielding requirements. This allows low-field MRI to be used in emergency departments, local offices, or lower-budget facilities. However, it is worth noting that low-field MRI still faces image quality limitations resulting from reduced signal strength [53]. In summary, a comprehensive approach to magnetic technologies used in MRI must go beyond superconducting electromagnets and also include classical resistive electromagnets and permanent magnet solutions, especially in the context of modern low-field and portable diagnostic systems.

Unlike imaging methods using ionizing radiation, MRI is a safe and non-invasive technique. However, caution is necessary for patients with ferromagnetic, electronic, or metal implants that may interact with a strong magnetic field and pose a risk. It is also important to minimize acoustic discomfort during the examination; therefore, both patients and staff are advised to use hearing protection, especially when working with gradients generating high noise levels [54,55].

#### 2.2.3. Application of MRI in Imaging Biological Structures

Magnetic resonance imaging (MRI) is unique in its ability to generate high-resolution images, enabling precise mapping of anatomical structures, including soft tissues, internal organs, and complex neural networks. Its non-invasive nature and lack of exposure to ionizing radiation make this technique the method of choice for diagnosing pathological changes such as cancerous tumors, both in animal models and in clinical patients. High imaging quality and the ability to repeat multiple examinations without biological risk make MRI widely used in monitoring disease progression and assessing therapeutic efficacy [56,57].

In pharmaceuticals and drug discovery research, MRI plays a key role in monitoring the distribution of active substances in tissues, assessing their impact on metabolic processes, and analyzing physiological parameters such as perfusion and blood flow dynamics. This data is extremely valuable in the design and optimization of pharmacological therapies, enabling precise determination of bioavailability, active compound release rate, and local tissue response to treatment. MRI also allows for non-invasive examination of the tumor microenvironment, including vascularization, degree of hypoxia and volume changes, which is important in the context of targeted therapies [58,59].

The development of advanced modalities such as functional magnetic resonance imaging (fMRI) and magnetic resonance spectroscopy (MRS) has significantly expanded the diagnostic and research capabilities of this technique. fMRI enables the observation of changes in blood oxygenation (BOLD) in response to neuronal activity, allowing for the analysis of the functional organization of the brain, its connectivity networks, and responses to sensory and cognitive stimuli. MRS, in turn, provides information on the biochemical composition of tissues, enabling the assessment of metabolite concentrations characteristic of metabolic disorders, neurodegenerative processes, and cancer. This makes this technique a valuable tool in both basic and preclinical research [40,60,61].

Optimization of magnetic field parameters, gradients, and RF pulse sequences enables the acquisition of high-contrast and highly detailed images. This allows MRI to detect even subtle morphological changes, such as microdamage in neural tissue resulting from trauma, inflammation, or the early stages of neurodegenerative processes. The ability to detect changes invisible in other imaging modalities makes MRI a key tool in modern diagnostics and the design of new therapeutic strategies.

#### 2.2.4. The Importance of Contrast Agents in MRI

Contrast agents play a key role in magnetic resonance imaging (MRI), enabling increased signal differences between individual tissues by selectively modifying the T1 and T2 relaxation times. Gadolinium chelates, such as Gd-DTPA or Gd-DOTA, are most commonly used. They shorten the T_1_ relaxation time, leading to increased signal intensity in T1-weighted sequences. This allows for the visualization of areas of increased vascular permeability, which is particularly important in the diagnosis of tumors, inflammatory lesions, and perfusion defects [62,63]. An alternative is superparamagnetic iron oxide nanoparticles (SPIONs), which primarily affect T2 relaxation and cause local signal intensity reduction in T_2_-weighted images. Their magnetic properties enable not only precise differentiation of anatomical structures but also monitoring biological processes and tracking the distribution of therapeutic molecules in real time, which is useful in research on drug nanocarriers and targeted therapies [64].

In pharmaceutical research, contrast agents are an invaluable tool for analyzing drug pharmacokinetics, enabling the assessment of their bioavailability, tissue distribution, and interactions with molecular targets. However, their potential adverse effects must be considered—gadolinium compounds, in particular, can exhibit nephrotoxicity and lead to nephrogenic systemic fibrosis in patients with renal failure. Therefore, in clinical practice, individual risk assessment and the use of minimally effective doses of the contrast agent are necessary [65].

In recent years, modern, biocompatible, and functionalized contrast agents based on nanoparticles have been intensively developed, combining high signal sensitivity with the possibility of selective molecular targeting. These structures have the potential to increase the safety of MRI diagnostics and improve the accuracy of pathological lesion detection, representing an important direction in the development of biomedical imaging. Table 2 below summarizes the main types of contrast agents used in MRI, their mechanisms of action, their effect on relaxation parameters, and key applications and limitations.

Manganese-based contrast agents have gained substantial attention due to their dual role as T1 enhancers and surrogates for calcium ions. Mn^2+^ can enter excitable cells via voltage-gated calcium channels, accumulating in proportion to cell activity. This feature enables activity-dependent imaging, which complements optical methods such as calcium fluorescence reporters. MRI has been successfully applied in neuronal circuit tracing, cardiac excitation mapping, pancreatic islet imaging, and tumor viability assessment. Importantly, the use of manganese aligns MRI with the molecular and functional sensitivity traditionally associated with fluorescence imaging, establishing MRI as a critical bridge for multimodal applications. Such agents transform MRI from a purely structural modality into a molecular imaging platform, paralleling the evolution seen in fluorescence imaging, where molecular specificity plays a central role [66,67]. As multimodal imaging gains importance in biomedical research, the integration of MRI with optical methods has increased significantly. Unlike fluorescence imaging, which provides molecular and cellular resolution, MRI offers deep anatomical context and functional readouts of whole organs. Innovative methods in this area include MRI-compatible fluorescence endoscopes, which enable minimally invasive bimodal imaging of the gastrointestinal and genitourinary systems, and bimodal nanoparticles designed to provide fluorescence contrast, often through the combination of fluorophores, high-conversion materials, or paramagnetic metals [68]. These hybrid systems are becoming increasingly sophisticated, supporting co-registration of optical and MRI datasets. Despite these advances, MRI still faces challenges similar to those discussed in fluorescence imaging. Limitations for high-field systems include long acquisition times, artifacts, and limited compatibility with optical equipment. High-dose toxicity remains a concern for manganese-based agents, fueling research into targeted delivery systems with improved safety. For ultra-low-field-of-view MRI, the reduced SNR and spatial resolution require advanced reconstruction algorithms and optimized coil design. However, ongoing advances in hardware, contrast chemistry, and hybrid control and measurement equipment continue to address these limitations, bringing MRI closer to seamless integration with optical imaging [69].

#### 2.2.5. Advantages and Challenges of MRI in Pharmaceutical and Clinical Research

Magnetic resonance imaging (MRI) is one of the most important tools in modern diagnostics and pharmaceutical research, offering a unique combination of non-invasiveness, high diagnostic efficiency, and safety. The lack of exposure to ionizing radiation makes this technique suitable for repeated measurements in both patients and animal models, which is important for long-term preclinical studies. Extremely high spatial resolution and excellent soft tissue contrast enable accurate assessment of therapy efficacy and the observation of subtle pathological changes at the structural and functional levels. Furthermore, the ability to record dynamic phenomena, such as the diffusion of therapeutic substances or changes in blood flow, allows for the analysis of physiological processes in real time [70].

Despite its numerous advantages, MRI also has significant limitations. The high costs of purchasing, operating, and maintaining MRI systems require specialized infrastructure and highly qualified personnel, limiting the availability of this technology in some research and clinical centers. Prolonged scanning sessions can be burdensome for patients suffering from claustrophobia or having difficulty maintaining stillness, potentially leading to motion artifacts. An additional challenge is the use of contrast agents, which can cause adverse reactions, including allergic reactions or nephrotoxicity, especially in individuals with renal impairment [71,72].

In preclinical studies, the limited availability of high-field MRI systems, which are essential for obtaining high-quality images in small animal models, remains a limitation. Image quality can also be compromised by magnetic field inhomogeneity and artifacts resulting from movement, breathing, or blood flow. To expand the range of diagnostic possibilities, hybrid systems are being developed that integrate MRI with other modalities, such as PET, allowing for the acquisition of complementary structural, functional, and metabolic information [73,74].

Recent reviews highlight the growing role of multimodal imaging techniques in nanomedicine. Combining MRI with other methods, such as PET or fluorescence, enables simultaneous monitoring of nanoparticle pharmacokinetics, biodistribution, and mechanisms of therapeutic action, providing real-time data on their efficacy and safety. Modern MRI-PET systems enable mapping of both deep anatomical structures and molecular interactions of nanoparticles, exceeding the capabilities of single imaging modalities [68].

MRI remains one of the most powerful tools in modern science and medicine, enabling comprehensive analysis of tissue structure and function based on resonance and relaxation phenomena. Combined with modern data analysis methods, including artificial intelligence algorithms, MRI has the potential for further development towards more precise, automated diagnostics and personalized medicine, supporting the selection of therapies tailored to individual patient needs [75,76].

### 2.3. Combination of Fluorescence and MRI

#### 2.3.1. The Concept of Multimodal Imaging

Multimodal imaging is a groundbreaking approach in medical diagnostics and preclinical research, combining complementary imaging techniques to obtain a more comprehensive, precise, and multidimensional view of biological processes. The integration of fluorescence and magnetic resonance imaging (MRI) is particularly valuable because it combines the high anatomical resolution of MRI with the exceptional molecular sensitivity of fluorescence. This allows for the simultaneous study of the location of anatomical structures and monitoring of processes occurring at the cellular and molecular levels. Fluorescence enables the detection of very low-intensity signals, often originating from single molecules, allowing for the analysis of protein–protein interactions, gene expression, metabolic changes, and the distribution of therapeutic compounds. MRI, on the other hand, provides non-invasive visualization of deep tissue structures, enabling the assessment of anatomy, organ function, and physiological parameters without the need for ionizing radiation [77,78,79,80]. The synergy of these techniques compensates for each other’s limitations. Fluorescence, despite its very high sensitivity, has a limited penetration depth, typically a few millimeters, preventing imaging of deeply located structures. MRI, on the other hand, offers excellent tissue penetration and high structural contrast but is characterized by lower molecular sensitivity. Combining both techniques enables the simultaneous analysis of biochemical changes and assessment of the anatomical context, which is particularly important in oncology, neurology, and cardiology [81].

In cancer tumor research, multimodal imaging enables the simultaneous assessment of the tumor’s biochemical characteristics—such as oxygen levels, biomarker expression, and metabolism—using fluorescence, and the precise determination of its location, shape, and size using MRI. Research indicates that this approach can increase diagnostic accuracy compared to single modalities, especially in the early stages of the disease [81,82].

Hybrid probes capable of simultaneously generating a fluorescent signal and MRI contrast play a key role in multimodal imaging. An example is nanoparticles containing gadolinium complexes and fluorophores emitting in the near-infrared (NIR) range, enabling precise correlation of data from both modalities and improving the quality of image reconstruction [82,83]. In clinical practice, multimodal imaging supports the development of personalized medicine, as it allows for more precise diagnosis and real-time monitoring of therapy effects. In research on cell therapies, such as stem cell transplantation, multimodality enables tracking of cell migration, survival, and integration within the patient’s body. Integrating data from different modalities, however, poses a challenge, requiring advanced image registration and analysis algorithms. In the future, the development of artificial intelligence technologies could significantly improve the efficiency and automation of multimodal analyses, making them a key tool in modern diagnostics and therapy [75,76,82,83].

#### 2.3.2. Technologies Combining Fluorescence and MRI

The integration of fluorescence and magnetic resonance imaging (MRI) in multimodal imaging systems relies on advanced technologies that enable the simultaneous recording of optical and magnetic signals through the use of specially designed hybrid probes. Nanostructures combining paramagnetic and fluorescent properties, such as superparamagnetic iron oxide nanoparticles (SPIONs) modified with organic fluorophores or quantum dots, play a key role. These nanoparticles shorten the T1 or T2 relaxation times in MRI while simultaneously emitting an intense fluorescent signal in the near-infrared (NIR) range, reducing interference with tissue autofluorescence and increasing detection accuracy [84,85,86]. Among the most promising solutions are liposomes and dendrimers containing gadolinium complexes with fluorescent dyes such as indocarbocyanines (e.g., ICG). They are characterized by high stability and biocompatibility, making them suitable for in vivo imaging with penetrations of approximately 1–2 cm, which is particularly important in preclinical studies. Studies have shown that nanoprobes of this type increase the signal-to-noise ratio (SNR) in tumor imaging by up to 5–10 times compared to classic monomodal contrast agents, significantly improving the quality and reliability of diagnostic data [86,87,88].

Platforms utilizing fluorine-19 (^19^F) in MRI are also an innovative direction of development. Perfluorocarbon molecules conjugated with fluorophores enable the acquisition of signals with distinct spectra, enabling multicolor imaging and precise separation of signals originating from different molecular structures or processes. This technology enhances the analytical capabilities of imaging at the cellular level, providing a valuable tool in pharmacokinetic studies and targeted therapies [89]. In parallel, hardware systems enabling the synchronous acquisition of fluorescence and MRI data are being developed. Modern solutions integrate optical elements with MRI magnets, reducing image registration errors and improving spatial and temporal consistency between modalities. At the molecular level, SNAP-Tag technology, based on the specific binding of hybrid probes to proteins, has proven to be a breakthrough. This allows for real-time monitoring of cell migration, proliferation, and fate—e.g., cancer or regenerative cells. Such systems are increasingly finding applications in theranostics, combining diagnostics with therapy, for example, by creating nanoparticles capable of therapeutic activation under the influence of light or a magnetic field [90].

However, the development of hybrid technologies is associated with a number of challenges, the most important of which concern the biocompatibility and safety of the probes. This includes minimizing the toxicity of heavy metal ions, limiting the potential accumulation of gadolinium, and improving the stability of the fluorescent signal in vivo. Further developments envisage the integration of fluorescence and MRI with additional modalities such as computed tomography (CT) and positron emission tomography (PET), leading to the development of trimodal or even multimodal imaging platforms. Although the construction of such systems is associated with high costs and significant technological complexity, their diagnostic potential can significantly expand the possibilities of biological analysis and contribute to a better understanding of complex molecular and anatomical processes [90,91,92].

#### 2.3.3. Examples of Hybrid Probes

**Figure 7:**
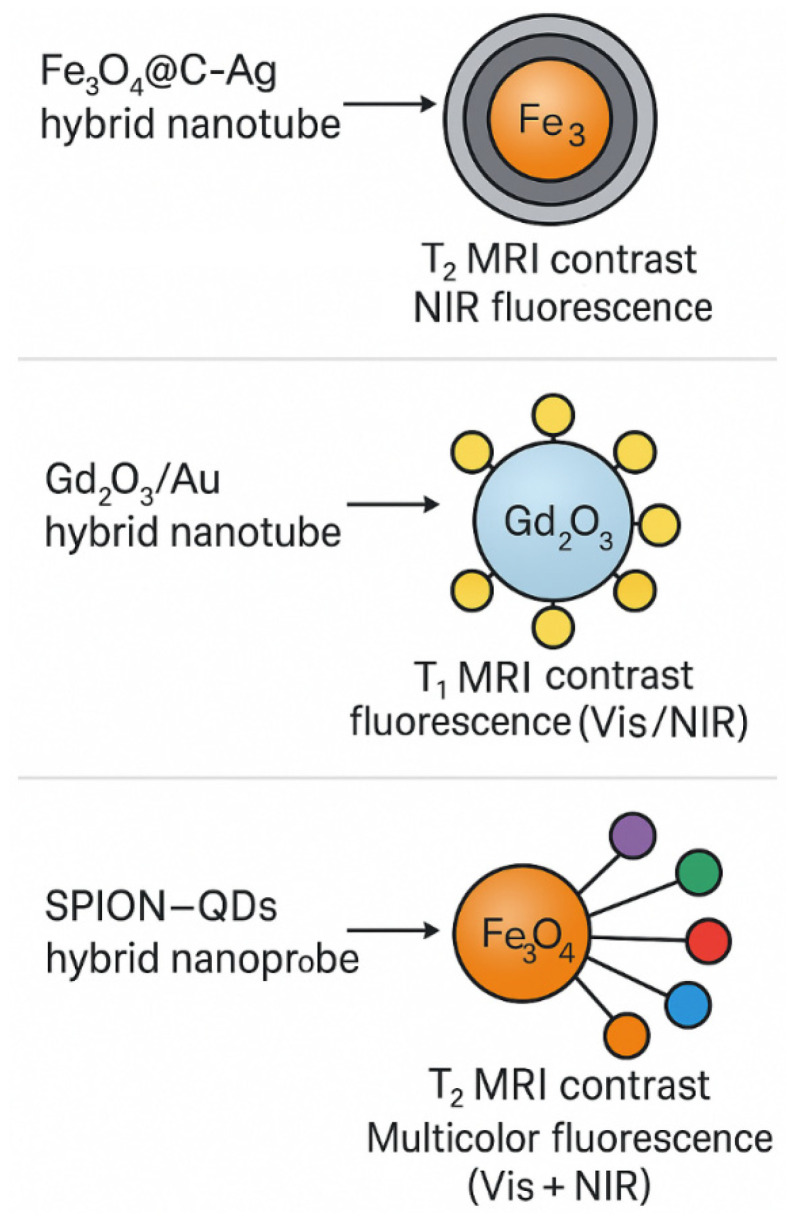
Schematic representation of hybrid multimodal imaging probes.

Examples of hybrid probes provide a key demonstration of the capabilities of technologies combining fluorescence and magnetic resonance imaging (MRI), enabling the simultaneous acquisition of molecular and anatomical information in preclinical and, in some cases, clinical models. Hybrid nanostructures combining light-emitting properties with magnetic features are currently one of the most dynamically developing areas of multimodal imaging [93,94]. One of the most advanced examples is a nanotube based on an Fe_3_O_4_ core with a carbon–silver coating (Fe_3_O_4_@C@Ag) (Figure 7). The superparamagnetic core generates T_2_-weighted contrast in MRI, while the silver–carbon layer is responsible for fluorescence emission in the near-infrared (NIR) range, minimizing interference from tissue autofluorescence. This probe has been used to image tumor vasculature in mouse models. It demonstrated high specificity of accumulation in tumor tissues, resulting from the enhanced permeability and retention (EPR) effect, and enabled the detection of vascular microstructures with a resolution below 100 μm—a level difficult to achieve using a single modality [94,95].

A second example is hybrid nanotubes based on gadolinium oxide and gold nanoparticles (Gd_2_O_3_/Au), synthesized by simultaneous co-precipitation using serum albumin (BSA) as a stabilizer. These nanostructures combine the paramagnetic properties of gadolinium (T_1_ contrast) with the plasmon fluorescence of gold, enabling simultaneous molecular detection and assessment of spatial signal distribution. In liver cancer models, these probes demonstrated the ability to selectively accumulate within the tumor following intravenous administration. NIR fluorescence confirmed high specificity of binding to apoptosis biomarkers, while MRI provided three-dimensional imaging of the tumor architecture. The achieved sensitivity for detecting apoptotic cells exceeded 95%, highlighting the theranostic potential of these structures [96].

The third group consists of magnetofluorescent probes based on iron nanoparticles (SPIONs) coupled to quantum dots (QDs), used in imaging carotid atherosclerotic plaques. These hybrids offer multicolor fluorescence in the visible and NIR ranges and T_2_ contrast in MRI, enabling multiparameter analysis of vascular lesions. In rabbit models, these probes enabled the detection of inflammatory foci in atherosclerotic plaques with high accuracy—estimated at approximately 90%. The fluorescent signal indicated the activity of macrophages associated with the inflammatory process, while MRI images allowed the assessment of vessel wall thickness and the degree of structural remodeling [97,98].

The figure presents a schematic overview of representative hybrid nanoprobes designed for multimodal imaging, integrating magnetic resonance contrast with fluorescent signal generation. The Fe_3_O_4_@C–Ag hybrid nanotube combines a superparamagnetic iron oxide core, responsible for T_2_-weighted MRI contrast, with a carbon–silver shell enabling near-infrared (NIR) fluorescence, enhancing molecular sensitivity and imaging depth. The Gd_2_O_3_/Au nanostructure merges gadolinium-based T_1_ MRI contrast with plasmon-enhanced fluorescence in the visible to NIR range provided by gold nanoparticles, supporting simultaneous molecular detection and anatomical visualization. The SPION–QDs hybrid nanoprobe incorporates Fe_3_O_4_ nanoparticles for T_2_ MRI contrast together with quantum dots that emit multicolor fluorescence (visible and NIR), enabling detailed assessment of vascular, inflammatory and oncologic lesions. Collectively, these examples illustrate how integrating magnetic and optical components within a single nanoplatform expands the diagnostic capabilities of multimodal imaging by improving spatial resolution, molecular specificity and quantitative assessment of disease processes.

The collected examples confirm that hybrid probes are extremely versatile multimodal imaging tools, with applications ranging from oncology to cardiology and inflammation diagnostics. Integrating surface functionalization with targeted ligands—such as antibodies, peptides, or aptamers—can further enhance the selectivity of probe accumulation in specific cell populations, paving the way for enhanced personalized diagnostics and therapy.

In recent years, particular attention has been directed toward hybrid probes incorporating second near-infrared window (NIR-II, 1000–1700 nm) fluorophores, which offer deeper tissue penetration, higher signal-to-background ratios, and improved spatial resolution compared with conventional NIR-I dyes. Emerging designs include both small-molecule NIR-II fluorophores with optimized emission and quantum yield, as well as inorganic and hybrid nanoplatforms that integrate NIR-II emission with additional functionalities such as magnetic or photothermal contrast. Preclinical and review studies indicate that such probes can support imaging of tumors—including central nervous system and bone malignancies—as well as inflammatory processes, enabling multimodal acquisition (e.g., NIR-II/MRI or NIR-II/photoacoustic imaging) alongside targeted phototheranostics. Incorporating NIR-II fluorophores into magnetofluorescent hybrid probes represents a natural extension of the technologies described above and significantly expands their translational potential [99,100,101,102].

In parallel with advances in NIR-II fluorophores and hybrid nanoplatforms, artificial intelligence and machine learning are increasingly employed in the design and optimization of multimodal probes. Monte Carlo-based simulations combined with AI algorithms facilitate prediction of imaging contrast and dosimetry for nanomaterials used in molecular cancer imaging, reducing the time required to identify optimal physicochemical parameters of probes [103]. Other studies emphasize “safety-by-design” approaches, integrating machine learning with ex vivo and in vivo assays to predict biocompatibility and immune interactions of metal–organic structures [104]. Machine learning models are also applied to forecast the composition of the protein corona on nanoparticle surfaces based on parameters such as size, zeta potential, and incubation conditions, which are essential determinants of biodistribution and targeting efficiency in vivo [105]. Furthermore, diagnostic systems combining magnetic nanoparticles (e.g., Fe_3_O_4_, MnO) with machine-learning-driven analysis in portable NMR platforms demonstrate that AI can support not only probe design but also intelligent signal interpretation in multimodal imaging and therapy monitoring [106].

#### 2.3.4. Challenges in Integrating Both Techniques

The integration of fluorescence and magnetic resonance imaging (MRI) in multimodal imaging systems presents a number of technical, biological, and regulatory challenges that limit the full potential of both methods. One key issue is the fundamental difference in the physical parameters of the two techniques. Fluorescence is characterized by very high molecular sensitivity, enabling the detection of single molecules, but its tissue penetration is limited to approximately 1–2 cm. MRI, on the other hand, provides unlimited imaging depth and excellent soft tissue contrast, but its low molecular sensitivity requires the use of relatively high-contrast agent concentrations, which can impact diagnostic safety [88]. Another significant challenge is the accurate registration and correlation of images from both modalities. Due to the different physical properties of the optical and magnetic signals, the use of advanced spatial alignment algorithms is necessary. Despite advances in image analysis, differences in positioning and tissue deformation remain possible, which can lead to quantification errors of up to 10–15%, especially in the case of dynamic in vivo processes such as perfusion or diffusion in tumor tissues [107,108]. Issues related to the toxicity and biocompatibility of hybrid probes also remain a significant limitation. Compounds containing heavy metals, such as gadolinium in T_1_ contrasts or iron in the form of SPION, can accumulate in organs, leading to immunological reactions, hepatotoxicity, or nephrotoxicity, especially in patients with impaired renal function. Gadolinium, as a heavy metal, carries a risk of toxicity both in situations of acute exposure and potential long-term retention in tissues. Gadolinium from MRI contrast agents can be deposited in various tissues, even in patients with normal renal function. Quantitative studies have shown that a small fraction of gadolinium can be retained in the liver for weeks after contrast administration and is correlated with the number of administrations, suggesting the possibility of long-term retention [109].

Pharmacokinetic studies indicate that Gd doses are typically eliminated within the first 24 h in individuals with normal renal function. However, residual gadolinium may persist for weeks or months, and measurable Gd concentrations in urine persist for more than three months after injection. Davies et al. also reported studies that observed prolonged gadolinium retention in bone for up to 8 years after administration, suggesting the existence of deep tissues with slow release kinetics [110].

Phototoxicity of fluorophores used at high doses or under intense illumination can also be problematic, limiting the duration of in vivo experiments. Additionally, many hybrid probes are characterized by limited stability in the physiological environment—structural degradation within a few hours shortens the diagnostic window and increases the risk of signal fluctuations [111,112]. Beyond the technical and biological aspects, economic and regulatory factors also pose barriers. Synthesis of multimodal probes requires advanced infrastructure and multi-stage optimization, which increases development costs. The process of approving new contrast agents for clinical use is subject to rigorous FDA and EMA regulations, including detailed toxicological and pharmacokinetic studies and long-term biocompatibility assessments. As a result, the clinical implementation path can take many years, further slowing the commercialization of new solutions [113].

Nanogels, polymeric structures, enable the encapsulation of gadolinium chelates or other metal agents, increasing their stability and limiting the release of free metal in the body. These carriers can serve as pH-sensitive platforms or those activated by the tumor microenvironment, improving contrast specificity and reducing side effects. Numerous studies are currently underway on the safety of these contrast agents, focusing on the risk of retention and toxicity. Linear agents, in particular, are currently under stricter scrutiny due to their higher propensity for deposition. In clinical practice, it is recommended to assess renal function before administering Gd and to use more stable macrocyclic agents to minimize the risk of free gadolinium release [114].

Modern nanotheranostic MRI platforms, in turn, integrate diagnostic and therapeutic functions in a single system, enabling not only the imaging of deep structures but also the controlled release of drugs and monitoring of therapy response. In such systems, multimodal contrasts combine the high anatomical contrast of MRI with the molecular sensitivity of optical techniques, creating opportunities for more effective tracking of the efficacy and biodistribution of nanotherapeutics in tumors. Nanotheranostic MRI-based systems enable simultaneous cancer diagnosis and therapy within a single nanoplatform. They utilize nanoparticles containing paramagnetic ions such as Fe, Gd, or Mn, which enhance MRI contrast and simultaneously act as drug carriers. MRI allows not only for precise tumor imaging but also for tracking nanoparticle distribution and monitoring treatment efficacy in real time. These nanoplatforms can be used in a variety of therapeutic strategies, including magnetic hyperthermia, photothermal and photodynamic therapy, chemodynamic therapy, immunotherapy, and ferroptosis induction. They can also be used in combination therapies that enhance treatment efficacy through synergistic effects. Proper design of nanoparticles for stability, biocompatibility, and the ability to selectively accumulate in tumor tissue is crucial [115].

In this context, magnetite nanoparticles are a significant development. Partial exchange of iron ions with manganese leads to improved magnetic properties and increased heat generation in an alternating magnetic field. The use of a double surfactant coating enables stable aqueous dispersions, reduces particle agglomeration, and improves their behavior under biological conditions, which is crucial for both effective hyperthermia and safe in vivo use. An increased SAR indicates higher heating efficiency, allowing for more effective destruction of cancer cells with lower doses and a reduced risk of damage to healthy tissue. At the same time, the demonstrated good biocompatibility of such nanoparticles confirms their potential for clinical applications. The combination of advanced nanoparticle design is consistent with the broader trend of developing intelligent nanotheranostics, which can significantly increase the efficacy and safety of cancer therapy [116].

### 2.4. Application of the Combination of Both Techniques in the Discovery of New Drugs

#### 2.4.1. Drug–Target Interaction Monitoring

An example of clinical and preclinical applications is hybrid probes conjugated with ligands specific for prostate membrane antigen (PSMA), used in the diagnosis and treatment of prostate cancer. They enable the detection of interactions with exceptionally high sensitivity, reaching below 1 nM, while offering submicron fluorescence imaging resolution and detailed anatomical context on MRI, including perfusion and diffusion changes within the tumor. This allows for more precise determination of tumor differentiation and identification of treatment-resistant areas [117].

The use of multimodal imaging combined with advanced computational methods, including deep generative models, improves the selection of new drug candidates and the analysis of drug–target interactions. As Zeng et al. [118] emphasize, “applications of deep generative models have already yielded new, partially optimized candidate candidates, in some cases in a shorter timeframe than conventional sequential approaches.” Collectively, available data indicate that integrating multimodal information and AI methods can significantly accelerate the drug discovery and development process [118,119].

In immuno-oncology, fluorescence–MRI probes enable real-time tracking of the interactions of checkpoint inhibitors, such as anti-PD-1 and anti-PD-L1 antibodies, with T cells. In mouse models, the use of hybrid magnetic-fluorescent nanoparticles targeting PD-L1 enabled precise mapping of receptor expression in tumors, ensuring high sensitivity and reliable spatial resolution of the detected signal. These observations support an iterative pharmacophore optimization process, enabling the assessment of the selectivity and affinity of newly developed molecules [118,119].

Challenges in monitoring drug–target interactions include the need to ensure high-probe selectivity to reduce nonspecific binding and to improve their stability in the physiological environment. Probe degradation shortens the diagnostic window and can lead to signal fluctuations, hindering accurate analysis of molecular processes. Therefore, increasing attention is being paid to fluorophores in the NIR-II range (1000–1700 nm), which are characterized by greater optical penetration and reduced light scattering, and to manganese (Mn^2+^)-based MRI contrast agents, which represent a safer and more biocompatible alternative to gadolinium-based contrast agents [120,121].

In addition to direct monitoring of drug–target interactions, multimodal fluorescence–MRI probes may also hold potential for investigating physicochemical processes fundamental to early drug development, such as tablet swelling [122], polymer hydration, and drug dissolution dynamics [123]—phenomena that have been extensively studied using MRI alone. While MRI offers high sensitivity to water mobility, hydration fronts, and structural changes within solid dosage forms, fluorescence could provide complementary molecular information by selectively reporting the spatial release profile or diffusion behavior of fluorescently labeled active pharmaceutical ingredients [124]. Such an integrated multimodal approach could, in principle, yield a more comprehensive understanding of dissolution mechanisms, particularly in complex formulations incorporating polymer matrices, multilayer systems, or nanocarrier-based delivery platforms. Although practical implementation would require addressing challenges such as limited light penetration in turbid pharmaceutical materials and maintaining photostability of fluorescent reporters, the combination of fluorescence and MRI represents a promising direction for advanced formulation analysis within the broader framework of drug discovery [125].

#### 2.4.2. Bioavailability and Pharmacokinetics Study

The use of hybrid fluorescence–MRI probes in bioavailability (BA) and pharmacokinetics (PK) studies is one of the most promising tools in contemporary experimental pharmacology. Combining two complementary techniques enables simultaneous monitoring of the distribution, metabolism, and elimination of active substances in living organisms, thus providing a comprehensive picture of the pharmacological fate of the drug with high spatial and temporal precision.

Analysis of doxorubicin bioavailability using liposomal nanoprobes allowed for the simultaneous monitoring of drug accumulation within the tumor (fluorescence) and changes in vascular perfusion (MRI). This enabled the identification of the influence of the heterogeneous tumor microenvironment on drug distribution and the identification of factors limiting therapy efficacy, such as poor blood–brain barrier penetration or variable vascular permeability in necrotic areas [126]. Hybrid probes also play a significant role in the evaluation of advanced drug delivery systems, such as polymer nanoparticles, ligand-targeted liposomes, and protein conjugates. In gene therapy studies, fluorescence–MRI probes demonstrated threefold higher siRNA accumulation in target cells compared to its free form, as confirmed by both the fluorescence signal and T_1_ contrast in MRI. This approach enables simultaneous quantitative assessment of the biodistribution, transport efficiency, and release efficiency of active substances in a specific microenvironment [127,128].

A key challenge is ensuring the probes’ stability in the biological environment, as enzymatic degradation, oxidation, and interactions with plasma proteins can shorten the probe’s duration of action to several hours.

#### 2.4.3. Disease Imaging in Preclinical Models

Hybrid fluorescence–MRI is widely used in preclinical models, enabling detailed analysis of disease progression in conditions such as cancer, neurodegenerative disorders, and cardiovascular pathologies. The combination of high fluorescence sensitivity with the deep penetration of magnetic resonance imaging allows for simultaneous monitoring of anatomical and molecular changes, making this technology one of the most promising translational tools in contemporary experimental medicine.

In oncology, hybrid probes such as superparamagnetic iron oxide nanoparticles conjugated with near-infrared fluorophores enable the detection of micrometastases less than 200 μm in diameter, achieving sensitivity up to ten times higher than that of conventional imaging techniques such as PET or CT. In breast cancer models, these probes allowed for the simultaneous tracking of anatomical changes (MRI) and the expression of biomarkers such as HER2 (fluorescence), enabling precise assessment of tumor heterogeneity with high precision [129,130,131].

In APP/PS1 transgenic models, a significant reduction in β-amyloid deposition was demonstrated following administration of monoclonal antibodies, confirming the usefulness of this modality in assessing the efficacy of new antineurodegenerative therapies [132,133].

In the field of cardiovascular diseases, hybrid probes enable the detection of inflammatory processes occurring in atherosclerotic plaques. Combining the fluorescent signal, which indicates macrophage activity, with MRI to assess the thickness and structure of the vessel wall allows for the determination of embolic risk with an accuracy of up to 85%. This enables early diagnosis of unstable atherosclerotic plaques, which can later lead to heart attack or stroke [134]. A key advantage of fluorescence–MRI in preclinical models is the ability to acquire complementary anatomical and molecular data in a single study session. This increases the reliability of results, significantly facilitates temporal analysis, and allows for real-time monitoring of disease dynamics. Technical challenges include optimizing acquisition time, necessary to minimize motion artifacts resulting from, among other things, animal breathing, as well as ensuring long-term probe biocompatibility, which is particularly important in experiments lasting several weeks.

#### 2.4.4. Examples and Achievements in Drug Development

Hybrid fluorescence–MRI has contributed to a number of significant advances in drug discovery and development, particularly in the areas of oncology, neurology, and targeted therapies. Combining high fluorescence sensitivity with deep MRI penetration enables simultaneous monitoring of molecular interactions, drug biodistribution, and tissue response, significantly accelerating therapeutic validation and iterative structural optimization of molecules. One of the most groundbreaking examples is the use of this technology in the design of tyrosine kinase inhibitors, such as imatinib, used to treat chronic myeloid leukemia (CML). Hybrid probes based on iron oxide nanoparticles conjugated with NIR fluorophores enabled monitoring of imatinib binding to BCR-ABL kinase in mouse models, revealing a 40% increase in drug efficacy after introducing specific chemical modifications. Fluorescence confirmed the specificity of the ligand–enzyme interaction, while MRI provided detailed data on the drug’s distribution within the bone marrow, supporting the process of precise pharmacophore refinement [135,136].

Another success is the development of hybrid nanoparticles used in gene therapy for cancers, including liver cancer. Fluorescence–MRI probes containing siRNA coupled with magnetic contrast agents and NIR fluorophores allowed for precise monitoring of the delivery of genetic material to cancer cells. Preclinical studies demonstrated threefold greater accumulation of siRNA in the tumor compared to the free form, while simultaneously inhibiting oncogene expression by approximately 80%. These effects accelerated the progression of the therapy to Phase I clinical trials, serving as an example of successful translation from nanotechnology to medicine [137].

In neurology, the use of hybrid probes has played a key role in the development of monoclonal antibodies targeting β-amyloid for the treatment of Alzheimer’s disease. In transgenic models, fluorescence–MRI demonstrated a reduction in amyloid deposits by approximately 25% after four weeks of therapy. Importantly, these changes correlated with measurable improvements in the animals’ cognitive function, highlighting the translational potential of this technology in neuroprotective drug design [138].

The presented examples clearly demonstrate that hybrid imaging techniques—combining fluorescence with MRI or other modalities—significantly improve the identification, validation, and early optimization of new therapeutic molecules. This multimodal approach simultaneously provides anatomical, molecular, and functional information, which, according to the literature, can significantly accelerate key stages of drug discovery and development compared to classical methods based solely on monomodal imaging or biochemical assays. Furthermore, in the field of neurodegeneration, the development of hybrid imaging protocols for small animals is emphasized as a significant factor in increasing the translational potential of new therapeutic strategies. Despite existing challenges—such as technology scalability, the complexity of probe production, and their cost—the dynamic progress in biodegradable nanocarriers and analysis automation creates a solid foundation for further development of this technology [139,140].

## 3. Conclusions

The combination of fluorescence and magnetic resonance imaging (MRI) in multimodal imaging is revolutionizing drug discovery by combining the high molecular sensitivity of fluorescence with the precise anatomical visualization of MRI. Hybrid probe-based technologies, including iron oxide nanoparticles, gadolinium complexes, polymer carriers, and NIR and NIR-II fluorophores, enable the tracking of drug–target interactions, the analysis of bioavailability and pharmacokinetics, and the monitoring of disease progression in preclinical models. In oncology, these techniques enable the detection of micrometastases and the assessment of tumor heterogeneity; in neurology, they enable the imaging of β-amyloid deposits; and in cardiology, they enable the identification of inflammation in atherosclerotic plaques. Combining fluorescence with MRI provides accuracy that cannot be achieved using single imaging modalities. Translational successes, such as the optimization of tyrosine kinase inhibitors and the development of nanoparticles for gene therapy, confirm that hybrid fluorescence–MRI can shorten drug development time by up to several years. The technology still faces numerous challenges, including probe toxicity, the complexity of their synthesis, difficulties in data synchronization, and regulatory constraints. At the same time, advances in nanotechnology, such as the development of biodegradable polymer carriers and gadolinium-free MRI contrast agents, as well as the application of artificial intelligence methods in data processing, offer real opportunities to overcome these barriers. The combination of fluorescence and MRI is establishing a new paradigm in biomedicine. Unlike classical methods that provide fragmented information about the structure, metabolism, or location of active substances, multimodal imaging allows for a coherent and dynamic image of biological processes. Integrating fluorescence sensitivity with MRI resolution enables the observation of molecular behavior within the context of the entire organism, in real time and without tissue disruption. Therefore, hybrid fluorescence–MRI is becoming a strategic platform in drug discovery, combining biophysics, pharmacology, materials science, and data analysis into a single, interdisciplinary research tool. The development of this technology, supported by device miniaturization, optimized regulatory procedures, and the application of machine learning, could lead to its introduction as a standard tool in personalized medicine.

## Figures and Tables

**Table 1:** Selected quantitative parameters describing fluorescence.

Parameter	Description	Importance in Molecular Analysis
Quantum efficiency (ΦF)	The ratio of the number of photons emitted to those absorbed	Determines the emission efficiency; influences the signal intensity
Fluorescence lifetime (τF)	Average excited state duration	Allows for analysis of the dynamics of molecular interactions
Stokes shift (Δλ)	The difference between the emission and excitation wavelengths	Provides information on energy losses and environmental impact
Photostability	Fluorophore resistance to bleaching	Key to long-term cell imaging

**Table 2:** Characteristics of the main types of contrast agents used in MRI.

	Mechanism of Action	Effect on Relaxation	Applications	Limitations
**Gadolinium chelates (Gd-DTPA, Gd-DOTA)**	Paramagnetism of Gd^3+^ ions	T1 shortening (increase in signal brightness)	Imaging of vessels, tumors, and inflammatory changes	Risk of nephrotoxicity, gadolinium accumulation
**SPION (Fe_3_O_4_, γ-Fe_2_O_3_)**	Superparamagnetism of iron nanoparticles	T2 shortening (signal intensity decrease)	Liver, spleen, lymphatic system	Potential toxicity, nanoparticle agglomeration
**Modern nanohybrid contrasts**	Hybrid metal–organic cores	Adjustable T1/T2 effect depending on composition	Molecular targeting, pharmaceutical research	Experimental research phase

## Data Availability

No new data were created or analyzed in this study. Data sharing is not applicable to this article.
